# Permanent Pacemaker Implantation After Transcatheter Aortic Valve Replacement: A Cost Analysis

**DOI:** 10.7759/cureus.5005

**Published:** 2019-06-26

**Authors:** Mansoor Ahmad, Jay N Patel, Brian L Loc, Sharath C Vipparthy, Chirag Divecha, Pablo X Barzallo, Minchul Kim, Timir Baman, Marco Barzallo, Sudhir Mungee

**Affiliations:** 1 Internal Medicine, University of Illinois College of Medicine at Peoria, Peoria, USA; 2 Cardiology, University of Illinois College of Medicine at Peoria, Peoria, USA

**Keywords:** permanent pacemaker, transcatheter aortic valve replacement, cost

## Abstract

Background

Transcatheter aortic valve replacement (TAVR) can be complicated with a complete atrioventricular block requiring permanent pacemaker (PPM) implantation. The cost of index hospitalization for such patients is higher than usual. However, the magnitude of this increased cost is uncertain. We have looked at our five-year TAVR experience to analyze the detailed cost for PPM implantation in TAVR.

Methods

This study is a retrospective analysis of patients undergoing TAVR at our tertiary care center from December 2012 to April 2018. The initial sample size was 449. We excluded patients with prior PPM or an implantable cardioverter defibrillator (37). Patients who had their procedure aborted or required a cardiopulmonary bypass (16) and those with missing data variables (14) were excluded. The final sample size was 382. The cost for admission was calculated as the US dollars incurred by the hospital. Cohort costs were categorized as a direct cost, which is patient based, and an indirect cost, which represents overhead costs and is independent of patient volume. Patients were divided into two groups based on the placement of PPM after TAVR. Chi-square test, t-test, and logistic linear regression were used for the statistical analysis.

Results

Of 382 patients, 19 (4.9%) required PPM after TAVR. Baseline variables, including age, gender, and BMI, were not statistically significant. The PPM group had a significantly longer intensive care unit (ICU) stay (48.6 hours vs. 36.7 hours; p<0.001) and total stay in the hospital (4.2 days vs. 3.4 days; p=0.047). PPM implantation after TAVR increased cost on an average of $10,213 more than a typical TAVR admission (p=0.04). The direct cost was also significantly high for the PPM group ($7,087; p=0.02). On detailed analysis, almost all major cost categories showed a higher cost for pacemaker patients when compared with control.

Conclusions

PPM implantation adds a significant cost burden to TAVR admissions.

## Introduction

Aortic stenosis is an insidious disease with a long latency period, followed by rapid progression after the appearance of symptoms, resulting in a high rate of death (approximately 50% in the first two years after symptoms appear) among untreated patients. Surgical aortic valve replacement (SAVR) reduces symptoms and improves survival in patients with aortic stenosis [1-3] and has historically been the standard of care. The first transcatheter aortic valve replacement (TAVR) was performed in 2002, and TAVR has, since then, grown in popularity [4-6].

When compared with SAVR, TAVR has demonstrated improved three-year clinical outcomes, including all-cause mortality, in the incidence of stroke and aortic valve hemodynamics [7]. However, these benefits can be offset by a much higher permanent pacemaker (PPM) implantation rate of 5%-25%, as SAVR has a PPM implantation rate of 1% per year [8]. The mechanism of atrioventricular (AV) block in TAVR is likely the direct injury to the His bundle, given its proximity to the membranous septum and native aortic valve [9-11]. Preexisting right bundle branch block (RBBB), low implantation, and the use of self-expanding valves (SEV) have been identified as common and consistent risk factors for PPM implantation across multiple studies [12-18]; a post-TAVR PPM implantation range from 6.0% for the Edwards SAPIEN (Edwards Lifesciences, Irvine, CA, US) balloon-expandable valve (BEV) to 25% for the Medtronic CoreValve (Medtronic, Inc., Minneapolis, MN, US), a self-expanding valve [19-22].

There is a paucity of data on the cost impact of PPM implantation after TAVR. The majority of data show a higher cost with PPM implantation post-TAVR [12]. However, studies have shown no significant cost difference after PPM implantation in the TAVR population [23]. One recent study has shown no significant difference in cost at one year after TAVR [23]. Review of the literature demonstrated a gap in knowledge regarding cost breakdown for this population. In this study, we have attempted to separate the cost into direct, indirect, and total costs for TAVR admission. In addition, we acquired the charged incurred by each department involved in patient care for TAVR admission, to elaborate on the areas that are impacted by PPM implantation in the TAVR population.

In addition to cost, we have looked at other outcomes, including the intensive care unit (ICU) stay, the total length of stay (LOS) in hospital, and 30-day readmission and mortality.

The abstract for this article was presented at the Society of Cardiovascular Angiography and Intervention Conference 2019 as a poster [24].

## Materials and methods

Patient population and study design

This is a retrospective chart review of 449 patients who received TAVR at OSF Saint Francis Medical Center between December 2012 and April 2018. First, patients with a prior pacemaker or implantable cardioverter defibrillator (37 patients) were excluded. Second, patients with an aborted procedure and those requiring a cardiopulmonary bypass or surgical conversion (16 patients) were excluded. Finally, patients with missing information for clinical variables (14 patients) were excluded. The final sample size was 382.

Institutional review board approval was obtained from the office of human research at the University of Illinois at Peoria, IL. Considering the retrospective nature of this study, the consent waiver was approved. All patients undergoing TAVR were deemed as intermediate or high risk for SAVR by the local cardiothoracic surgery team based on the Society of Thoracic Surgeons (STS) score.

Clinical, electrocardiographic, and echocardiographic data were extracted retrospectively, and every patient had a baseline electrocardiogram (EKG) and echocardiogram done before TAVR. The clinical variables studied included age, gender, body mass index (BMI), STS score, history of hypertension, diabetes, prior myocardial infarction, heart failure with different New York Heart Association functional classes (NYHA Class), atrial fibrillation or flutter, smoking, chronic lung disease, and renal disease requiring dialysis.

Echocardiographic variables included left ventricular internal diameter measured at systole and diastole (LVIDs/LVIDd) and ventricular septal wall thickness.

Outcome comparison

The primary outcomes included PPM implantation after TAVR. In addition, we looked at hours of stay in the ICU, hospital length of stay (LOS) in days, and readmission at 30 days after TAVR.

Secondary outcomes comprised cost differences between the two groups. We calculated the direct, indirect, and total cost of TAVR admission.

Cost categories

The direct cost was the cost incurred by individual patient care; it varied with patient volume. It was a combination of "direct fixed" and "direct variable cost." Direct fixed costs did not change with patient volume. It included fixed labor, e.g. salaries and wages; fixed benefits of staff, e.g. health insurance, Federal Insurance Contributions Act (FICA), and 401k; and fixed purchase services, e.g. maintenance contracts, pharmaceuticals, equipment maintenance, and offset expenses. Direct variable costs were a combination of labor and benefits and implants, e.g. TAVR valves, pharmacy, blood supply, lab supplies, repair, and maintenance, and would be higher for complex patients and those with a longer stay.

Indirect costs covered the overhead cost allocated to each case; these costs are not volume-sensitive and cannot be impacted at the bedside, e.g. facility costs, housekeeping, maintenance, and information technology. Total cost was a combination of both direct and indirect costs.

Statistical analyses

Patients were divided into groups based on PPM status. Baseline characteristics and clinical data were compared among groups. Continuous data were represented as mean ± SD and categorical data as proportions. The t-test was used to compare continuous variables and Chi-square tests for categorical variables.

Adjusted statistical analyses were conducted for clinical and economic outcomes. For ICU hours and length of stay, a generalized linear model with log link and Poisson distribution was used. For the cost outcome (direct cost, indirect cost, and total cost), the generalized linear model with log link and gamma distribution was used. All cost variables were inflated to the 2018 US dollar using the Inpatient Hospital Service Consumer Price Index (CPI).

The key covariate is a PPM status variable. Common covariates for adjusted analysis included age, gender (male), smoking status, Society of Thoracic Surgeons (STS) score, body mass index (BMI), left ventricular internal diameter systolic (LVIDs), left ventricular internal diameter diastolic (LVIDd), septal wall thickness, valve type, valve size, prior NYHA functional class, chronic lung disease, diabetes, dialysis, prior myocardial infarction (MI), prior two-week diagnosis of heart failure, hypertension, atrial fibrillation/flutter, and conduction defect. For logistic analysis of the PPM outcome, the following variables were omitted due to collinearity: valve size, access type, and dialysis.

All calculations were performed using STATA 12 (STATA Corp, Texas, US) and a p-value of less than 0.05 was considered statistically significant.

## Results

Baseline characteristics

A total of 382 patients were included in this study (Table 1). Of these patients, PPM was seen in 19 (4.97%) patients. When comparing the two groups, there was no significant difference between age (PPM: 81.6 vs. non-PPM: 80.5; p=0.564) and STS score (PPM: 7.5 vs. non-PPM: 6.8; p=0.588). Gender was also not statistically different between the two groups (males: PPM: 57.9% vs. non-PPM: 51.2%; p=0.572).

**Table 1:** Baseline characteristics * Chi-square test for categorical variables and t-test for continuous variable Standard Deviation (SD) and proportion (%) are by the columns. Society of Thoracic Surgeons (STS), left ventricular internal diameter systolic (LVIDs), left ventricular internal diameter systolic (LVIDd), heart failure (HF), myocardial infarction (MI)

Variables	All sample (N=382)	PPM (N=19)	No PPM (N=363)	
Continuous variables	Mean (SD)	Mean (SD)	Mean (SD)	P value*
Age	80.5 (8.5)	81.6 (8.7)	80.5 (8.5)	0.564
STS score	6.9 (5.1)	7.5 (6.4)	6.8 (5.1)	0.588
LVIDs	3.2 (0.8)	3.0 (0.9)	3.2 (0.8)	0.367
LVIDd	4.6 (0.7)	4.7 (0.9)	4.6 (0.7)	0.606
Categorical variables	# of sample (proportion)	# of sample (proportion)	# of sample (proportion)	P value*
Male	197 (51.6%)	11 (57.9%)	186 (51.2%)	0.572
Smoker	21 (5.5%)	2 (10.5%)	19 (5.2%)	0.324
Valve type				0.172
Sapien	44 (11.5%)	1 (5.3%)	43 (11.8%)	
Sapien XT	69 (18.1%)	1 (5.3%)	68 (18.7%)	
Sapien 3	269 (70.4%)	17 (89.4%)	252 (69.4%)	
Valve size				0.071
20 mm	14 (3.6%)	0 (0.0%)	14 (3.8%)	
23 mm	141 (36.9%)	5 (26.3%)	136 (37.5%)	
26 mm	153 (40.1%)	6 (31.6%)	147 (40.5%)	
29 mm	74 (19.4%)	8 (42.1%)	66 (18.2%)	
Body Mass Index				0.083
Underweight (<25)	95 (24.9%)	3(15.8%)	92 (25.3%)	
Normal (25~ <30)	133 (34.8%)	3 (15.8%)	130 (35.8%)	
Overweight (30~ <35)	81 (21.2%)	7 (36.8%)	74 (20.4%)	
Obesity (>=35)	73 (19.1%)	6 (31.6%)	67 (18.5%)	
Septal wall thickness				0.283
<1.1	77 (20.2%)	2 (10.5%)	75 (20.7%)	
>=1.1	305 (79.8%)	17 (89.5%)	288 (79.3%)	
Access type				0.915
Femoral	331 (86.6%)	16 (84.2%)	315 (86.8%)	
Trans Aortic	23 (6.0%)	2 (10.5%)	21 (5.8%)	
Trans Apical	23 (6.0%)	1 (5.3%)	22 (6.1%)	
Trans Iliac	3 (0.8%)	0 (0.0%)	3 (0.8%)	
Subclavian	2 (0.5%)	0 (0.0%)	2 (0.5%)	
Prior-NYHA 2 category				0.913
I-II	57 (14.9%)	3 (15.8%)	54 (14.9%)	
III-IV	325 (85.1%)	16 (84.2%)	309 (85.1%)	
Chronic lung disease				0.871
None	212 (55.5%)	10 (52.6%)	202 (55.7%)	
Mild	83 (21.7%)	4 (21.1%)	79 (21.7%)	
Moderate	57 (14.9%)	4 (21.1%)	53 (14.6%)	
Severe	30 (7.8%)	1 (5.2%)	29 (8.0%)	
Diabetes	157 (41.1%)	7 (36.8%)	150 (41.3%)	0.699
Dialysis	14 (3.7%)	0 (0.0%)	14 (3.9%)	0.383
Prior MI	114 (29.8%)	6 (31.6%)	108 (29.7%)	0.865
Prior HF	105 (27.5%)	4 (21.1%)	101 (27.8%)	0.519
Hypertension	353 (92.4%)	15 (78.9%)	338 (93.1%)	0.023
Atrial Fibrillation/Flutter	140 (36.6%)	8 (42.1%)	132 (36.4%)	0.613
Conduction Defect	198 (51.8%)	16 (84.2%)	182 (50.1%)	0.004

Clinical variables

The non-PPM group saw comparatively more patients with hypertension as compared to the PPM group (PPM: 78.9% vs. non-PPM: 93.1%; p=0.023). The percentage of patients with conduction defect was significantly higher in PPM patients (PPM: 84.2% vs. non-PPM: 50.1%; p=0.004). No other clinical variable was statistically significant.

Regression analysis

After adjusting for other clinical variables, logistic regression (Table 2) showed that patients with larger LVIDs were less likely to receive PPM than those with smaller LVIDs (OR: 0.20, CI: 0.04 - 0.91, p=0.038). No other variable was statistically significant.

**Table 2:** Adjusted logistic regression result (outcome: PPM, n=368) The following variables were omitted due to collinearity: valve size, access type, and dialysis. Valve size of 20 mm (14 observations) was omitted due to no observation in the PPM group. Society of Thoracic Surgeons (STS) score, body mass index (BMI), left ventricular internal diameter systolic (LVIDs), left ventricular internal diameter diastolic (LVIDd), myocardial infarction (MI), heart failure (HF)

Covariates	Odds Ratio	P value	95% Confidence Interval
Age	1.05	0.199	(0.97, 1.15)
Male	0.24	0.152	(0.03, 1.68)
Smoker	5.14	0.148	(0.55,47.24)
STS score	1.04	0.507	(0.92, 1.17)
BMI (Ref: Normal)			
Underweight	0.85	0.865	(0.13, 5.55)
Overweight	3.77	0.104	(0.76, 18.65)
Obese	5.57	0.050	(1.00, 31.05)
LVIDs	0.20	0.038	(0.04, 0.91)
LVIDd	4.38	0.077	(0.85, 22.55)
Septal wall thickness >=1.1 (Ref: <1.1)	1.21	0.831	(0.20, 7.06)
Valve type (Ref: Sapien)			
Sapien XT	1.24	0.890	(0.05, 27.49)
Sapien 3	4.69	0.232	(0.37, 59.21)
Valve size (Ref: 23mm)			
26 mm	2.19	0.347	(0.05, 27.49)
29 mm	7.07	0.090	(0.37, 59.21)
Prior NYHA III-IV	0.77	0.746	(0.16, 3.66)
Chronic lung disease (Ref: None)			
Mild	0.88	0.869	(0.19, 4.00)
Moderate	1.29	0.745	(0.27, 6.20)
Severe	0.32	0.430	(0.01, 5.35)
Diabetes	0.52	0.290	(0.15, 1.74)
Prior MI	1.18	0.787	(0.35, 3.91)
Prior HF	1.01	0.986	(0.21, 4.73)
Hypertension	0.25	0.091	(0.05, 1.24)
Atrial Fibrillation/Flutter	1.11	0.848	(0.35, 3.57)
Conduction Defect	3.72	0.057	(0.96, 14.38)
Constant	1.9E-06	0.020	(7.37E-10, 0.16)

Multivariable analysis

Patients receiving PPM stayed an average of 12 hours more in the ICU than the non-PPM group (48.6 vs. 36.7 hours; p<0.001) (Table 3). Length of stay in the hospital, although longer for the PPM group, was not statistically significant (PPM: 4.21 vs. non-PPM: 3.40; p=0.116).

**Table 3:** Multivariable analysis result (n=382) All costs were converted to 2018 US dollar using the Inpatient Hospital Service Consumer Price Index (CPI). Covariates included pacemaker, age, male, smoking status, STS score, BMI, LVIDs, LVIDd, septal wall thickness, valve type, valve size, access type, prior NYHA, chronic lung disease, diabetes, dialysis, prior MI, prior two-week HF, hypertension, atrial fibrillation/flutter, and conduction defect. ^1 ^Generalized linear model (GLM) with log link and Poisson distribution adjusting for the above covariates. ^2^ Generalized linear model (GLM) with log link and gamma distribution adjusting for the above covariates. Permanent pacemaker (PPM), body mass index (BMI), New York Heart Association functional (NYHA), Society of Thoracic Surgeon (STS), heart failure (HF), myocardial infarction (MI)

Outcomes	PPM (N=19) Mean (95% CI)	No PPM (N=363) Mean (95% CI)	Difference Mean (95% CI)	P value
ICU hours^1^	48.6 (45.2, 52.0)	36.7 (36.1, 37.3)	11.9 (8.4, 15.3)	<0.001
Length of stay^1^	4.21 (3.23, 5.18)	3.40 (3.21, 3.59)	0.80 (-0.19, 1.80)	0.116
Total cost^2^	$81,701 (72162, 91240)	$71,487 (69660, 73315)	$10,213 (453, 19973)	0.040
Direct cost^2^	$53,862 (47862, 59862)	$46,774 (45636, 47912)	$7,087 (952, 13223)	0.024
Pharmacy	$1,029 (290, 1768)	$860 (664, 1056)	$168 (-554, 891)	0.648
Blood	$52 (-42, 148)	$827 (-398, 2052)	-$774 (-2003, 454)	0.217
Laboratory	$601 (414, 788)	$632 (580, 685)	-$31 (-219, 156)	0.742
Room	$5,568 (3326, 7810)	$4,001 (3609, 4393)	$1,566 (-684, 3817)	0.173
Supply	$315 (-24, 654)	$193 (120, 266)	$121 (-198, 442)	0.457
Therapy	$980 (531, 1429)	$779 (682, 876)	$201 (-245, 647)	0.377
Imaging	$487 (284, 690)	$430 (388, 473)	$57 (-149, 263)	0.588
Miscellaneous cost	$46,076 (42838, 49314)	$39,205 (38604, 39806)	$6,871 (3561, 10180)	<0.001
Indirect cost^2^	$27,850 (24177, 31523)	$24,709 (23991, 25427)	$3,141 (-622, 6904)	0.102

Cost analysis

PPM implantation significantly increased the total and direct cost for TAVR admission (Table 3). The average total cost for the PPM group was higher by $10,213 ($81,701 vs. $71,487; p=0.040). Direct cost was higher for the PPM group by $7,087 ($53,862 vs. $46,774; p=0.024). Indirect cost, although higher for the PPM group, was not statistically different ($27,850 vs. $24,709; p=0.102).

Looking at the breakdown of costs (Table 3), the PPM group incurred a higher cost for almost every subcategory, except for blood products and laboratory use. “Miscellaneous costs” were significantly higher for the PPM group by $6,871 ($46,076 vs. $39,205; p<0.001). These miscellaneous costs (Table 4) comprised services received by patients during admission that were specific to the patient’s co-morbidities and usually not directly related to the TAVR procedure. These do include electrophysiology services, which were clearly involved with all patients in the PPM group.

**Table 4:** Miscellaneous costs Peripherally inserted central catheter (PICC)

Miscellaneous Cost
Surgery - General (Major)
Electrophysiology
Gastrointestinal Services
Pulmonary Intervention Lab
Psychiatric Emergency Care
Recovery Room Services
IV Nutritional Support
Emergency Services
ER Physicians
Electroencephalography
Hematology services
Urology Clinic
Pulmonary Function Services
PICC Line Team

Readmission and mortality

PPM implantation increased the odds of readmission rate at 30 days (OR: 1.19, CI: 0.28 - 4.95, p=0.802), however, it was not statistically significant (Table 5). We could not calculate 30-day mortality due to the perfect collinearity between PPM and 30-day mortality.

**Table 5:** Adjusted logistic regression result (n=382) Note: Covariates included pacemaker, age, male, smoking status, Society of Thoracic Surgeons (STS) score, body mass index (BMI), left ventricular internal diameter systolic (LVIDs), left ventricular internal diameter systolic (LVIDd), septal wall thickness, valve type, valve size, prior New York Heart Association (NYHA), chronic lung disease, diabetes, dialysis, prior myocardial infarction (MI), prior two-week heart failure, hypertension, atrial fibrillation/flutter, and conduction defect. Access type was excluded due to multicollinearity. NA: not available ^1^ 30-day mortality was not available for analysis due to perfect collinearity between 30-day mortality and PPM.

Outcomes	Odds Ratio	P value	95% Confidence Interval
30-day readmission	1.19	0.802	(0.28, 4.95)
30-day mortality^1^	NA		

## Discussion

Transcatheter aortic valve replacement has proven itself as a safe alternative to surgical valve replacement in intermediate to high-risk patients with symptomatic aortic stenosis. Post-TAVR conduction abnormalities needing pacemaker placement are well-documented and, furthermore, have been described with greater frequency in TAVR with an incidence ranging from 5%-22% [25]. The proliferation of TAVR has increased the need for pacing post-implantation, with literature to date focusing on factors predicting the need for post-implant device therapy, including anatomical factors, type of prosthesis, and underlying conduction abnormalities [26]. Device therapy in this setting has been previously associated with increased length of stay, increased mortality, and, as our study more clearly highlights, increased cost [27].

Our facility exclusively utilizes the balloon expandable delivery systems of the Edwards Lifesciences Sapien line of devices, for which prior studies have identified a lower risk of post-implant conduction defects (around 5%) when compared to self-expanding device delivery systems (around 12%-39%) [28]. Our cohort compared well to previously published cohorts, with 19 patients (4.97%) necessitated pacing therapy [29]. Baseline characteristics between study groups were non-significant based on t-test and chi-squared testing, with the exception of underlying conduction abnormalities. Our study was not adequately powered for identifying independent predictive variables. However, logistic regression analysis of our cohort demonstrated the statistical significance of one observed variable, showing that patients with larger LVIDs were less likely to need PPM implantation. Baseline conduction defects neared statistical significance (p=0.057). Lastly, there was no significant difference in 30-day readmission rates.

The primary outcomes of our study met statistical significance for increased intensive care stay and total cost in the pacemaker cohort. First, ICU stay, on average, was 11.9 hours longer than that of the non-pacemaker cohort (p=<0.001, CI 8.4-15.3). Second, post-TAVR PPM implantation was associated with an increased cost of $10,213 (p=0.040) when indexed to the 2018 US dollar using the Inpatient Hospital Service Consumer Price Index (CPI). These compared well to the previously quoted figures of $6620-$11,885 in prior publications [25,27].

Our trial was novel in that it utilized itemized facility costs for the identification of factors leading to increased cost. Our facility divided costs based on direct patient care (including pharmacy, blood, laboratory, room, supplies, therapy, imaging, and miscellaneous costs) and indirect cost. Of these categories, direct patient care and miscellaneous costs met statistical significance (p=0.024 and <0.001, respectively). The direct cost was identified to be $7,087 more in the PPM cohort. The miscellaneous cost was $6,871 greater in the PPM cohort, indicative of the involvement of the electrophysiology service and ancillary staff required for a second procedure (Table 3). Based on our cost analysis, charges related to facility overhead costs were not statistically significant. Previously, it has been postulated that increased length of stay and increased utilization of finite facility resources lead to significant cost increases [30], however, conversely, our study demonstrated the primary drivers are procedure-related costs of the PPM implantation with a relatively fixed overhead cost.

Strengths and limitations of the study

Our calculations have used cost, indexed to the 2018 US dollar using the Inpatient Hospital Service CPI, which is inflation-adjusted for the study period. The detailed cost of direct and indirect expenses has not been extensively described in the literature for PPM implantation in the TAVR population.

The limitations of our study relate primarily to its limited applicability to other implantation centers based on regional and facility variation in costs and protocols related to post-TAVR conduction abnormalities. In addition, the retrospective nature of this study has inherent limitations. Our study was not adequately powered for the identification of independent predictive variables. Thirty-day mortality could not be calculated because of collinearity in the data, which is another limitation of this study.

## Conclusions

This study is the first to date that focused on identifying specific cost drivers related to post-transcatheter aortic valve replacement pacemaker implantation. The increased cost in our cohort was primarily attributed to procedural costs directly related to pacemaker implantation. However, facility utilization costs did not demonstrate significance. The goal of this study is to spur further research on cost reduction given the continued proliferation of TAVR.
